# Host-associated microbiomes drive structure and function of marine ecosystems

**DOI:** 10.1371/journal.pbio.3000533

**Published:** 2019-11-11

**Authors:** Laetitia G. E. Wilkins, Matthieu Leray, Aaron O’Dea, Benedict Yuen, Raquel S. Peixoto, Tiago J. Pereira, Holly M. Bik, David A. Coil, J. Emmett Duffy, Edward Allen Herre, Harilaos A. Lessios, Noelle M. Lucey, Luis C. Mejia, Douglas B. Rasher, Koty H. Sharp, Emilia M. Sogin, Robert W. Thacker, Rebecca Vega Thurber, William T. Wcislo, Elizabeth G. Wilbanks, Jonathan A. Eisen

**Affiliations:** 1 Genome and Biomedical Sciences Facility, University of California, Davis, Davis, California, United States of America; 2 Smithsonian Tropical Research Institute, Balboa, Ancon, Republic of Panamá; 3 Centre for Microbiology and Environmental Systems Science, Department of Microbial Ecology, University of Vienna, Vienna, Austria; 4 LEMM, Laboratory of Molecular Microbial Ecology, Institute of Microbiology Paulo de Góes, Federal University of Rio de Janeiro (UFRJ), Rio de Janeiro, Brazil; 5 IMAM-AquaRio–Rio de Janeiro Aquarium Research Center, Rio de Janeiro, Brazil; 6 Department of Nematology, University of California, Riverside, Riverside, California, United States of America; 7 Tennenbaum Marine Observatories Network, Smithsonian Environmental Research Center, Edgewater, Maryland, United States of America; 8 Centro de Biodiversidad y Descubrimiento de Drogas, Instituto de Investigaciones Científicas y Servicios de Alta Tecnología (INDICASAT AIP), Panamá, Republic of Panamá; 9 Center for Ocean Health, Bigelow Laboratory for Ocean Sciences, East Boothbay, Maine, United States of America; 10 Department of Biology, Marine Biology, and Environmental Sciences, Roger Williams University, Bristol, Rhode Island, United States of America; 11 Max Planck Institute of Marine Microbiology, Bremen, Germany; 12 Department of Ecology and Evolution, Stony Brook University, Stony Brook, New York, United States of America; 13 Department of Microbiology, Oregon State University, Corvallis, Oregon, United States of America; 14 Department of Ecology, Evolution, and Marine Biology, University of California, Santa Barbara, Santa Barbara, California, United States of America; 15 Department of Evolution and Ecology, University of California, Davis, Davis, California, United States of America; 16 Department of Medical Microbiology and Immunology, University of California, Davis, Davis, California, United States of America

## Abstract

The significance of symbioses between eukaryotic hosts and microbes extends from the organismal to the ecosystem level and underpins the health of Earth’s most threatened marine ecosystems. Despite rapid growth in research on host-associated microbes, from individual microbial symbionts to host-associated consortia of significantly relevant taxa, little is known about their interactions with the vast majority of marine host species. We outline research priorities to strengthen our current knowledge of host–microbiome interactions and how they shape marine ecosystems. We argue that such advances in research will help predict responses of species, communities, and ecosystems to stressors driven by human activity and inform future management strategies.

## Abstract 2

The significance of symbioses between eukaryotic hosts and microbes extends from the organismal to the ecosystem level and underpins the health of Earth’s most threatened marine ecosystems. This Perspective article outlines research priorities to strengthen our current knowledge of host-microbiome interactions, to help predict responses to anthropogenic stressors and to inform future management strategies.

## Introduction

Within the vast and dazzling biological diversity that inhabits the world’s oceans, it would be challenging to find a eukaryotic organism that does not live in close relationship with a microbial partner. Such symbioses, i.e., persistent interactions between host and microbe in which none of the partners gets harmed and at least one of them benefits (Box 1), are ubiquitous from shallow reefs to deep-sea hydrothermal vents. Studies on corals [1], sponges [2], and mollusks [3–5] have revealed some of the profoundly important symbiotic roles microbes play in the lives of their hosts. These studies, however, have tended to focus on a small number of specific microbial taxa. In contrast, most hosts retain groups of many hundreds of different microbes (i.e., a microbiome [6,7]), which themselves can vary throughout the ontogeny of the host and as a result of environmental perturbations [8–10]. It is now clear that, rather than host-associated microbes functioning independently, complex multi-assemblage microbiomes have major impact on the fitness and function of their hosts. Studying these complex interactions and biological outcomes is difficult, but if we wish to truly understand the origin and evolution of organisms and populations and the structure and function of communities and ecosystems, we must advance our understanding of symbioses in host–microbiome systems [11,12].

**Box 1. Terminology used to discuss communities of microbes and their interactions with hosts.**

Following are key terms and concepts that we use in this paper (see also [7]).

**Microbiome and microbiota**

We use “microbiome” to refer to a community of microbes (organisms too small to see without the aid of a microscope) found at a specific place and/or a specific time. We avoid using the term “microbiota,” which has a complicated history (see [7]). Ideally, the place and/or time should be specified when discussing the microbiome. For example, “the seagrass microbiome” is the total of microbial communities found in association with seagrass, and “the seagrass root microbiome” would be those microbial communities found in/on the roots of seagrass. We also consider host-associated microbiomes broadly to include any and all kinds of microbes (e.g., bacteria, archaea, microbial eukaryotes, or viruses), which can be transient or persistent, and have variable functional impacts from beneficial to unimpactful to detrimental. Microbiomes can inhabit the external and internal surfaces of virtually every eukaryote, from microscopic unicellular diatoms to macroscopic organisms such as kelp, coral, seagrass, cephalopods, and vertebrates [8].

**Symbiosis**

We use “symbiosis” here in the broad sense, meaning a persistent relationship between two or more organisms in which at least one of them benefits. Symbioses come in three subcategories: in “mutualism,” both or many partners benefit; in “parasitism,” one partner benefits and the other(s) is/are harmed; in “commensalism,” at least one partner benefits and the other(s) is/are unaffected. In many discussions of microbes or microbial communities living in and on a host organism, it is frequently assumed that the microbe is benefitting in some way, and the question then becomes, “What is the effect on the host?” If the host benefits, this is a mutualism; if the host is unaffected, this is a commensalism; and if the host is harmed, this is a parasitism. It is important to note that these categories are fluid in that the type of interaction between two species is often conditional and depends on many factors including genotype (of all partners), environmental conditions, and developmental stage, among others.

**Health status and microbiomes**

Much of the work on host-associated microbiomes revolves around whether the community of microbes in some way affects the health status of the host [87]. In some cases, researchers have used terms like “healthy” or “dysbiotic” or “optimal” to describe a particular microbiome (e.g., of one host individual at one time) or pattern of change documented among particular groups. Although these terms can sometimes be useful in general discussions of microbiomes, they are hard to define quantitatively or apply and therefore more likely to confuse than to illuminate in practice. For example, an “optimal” microbiome could vary between individuals and across environmental conditions. Similarly, there could be numerous and equivalent alternative microbiome states, each of which could be referred to as “healthy,” which include transient or permanent neutral members and/or active symbiotic players. The inverse of a healthy association is “dysbiosis,” often suggested to be any change in the composition and/or variability of a microbial community that can cause any negative impact on the host. Generally, we believe such terms need to be used with extreme caution and to be clearly and quantitatively defined when in practice (see a useful discussion of this topic in [91]).

**Ecosystem functions**

“Ecosystem functions or processes” are generally considered to be aggregate fluxes of energy or materials [14]. Ecosystem function has also sometimes been defined as the joint effects of all processes, including fluxes of energy and chemical compounds, that sustain an ecosystem over time and space through biological activities [92]. Generally, ecosystem functioning depends disproportionately on a small subset of species in the system. These include particularly “foundation species” (dominant sessile invertebrates, plants, or algae that provide physical structure and have a strong role in structuring the community [93]) and “keystone species,” (taxa that have a large effect on other species that is disproportionate to their own relatively low abundance and that, if they were removed, would drastically change the ecosystem). “Resilience” is the capacity of an ecosystem to respond to a perturbation or disturbance by recovering quickly. Another form of response is “evolutionary adaptation,” in which species change genetically to adapt to a new environment. Over several generations and through the process of natural selection, physical and behavioral features of organisms may adapt to function better in the new environment. If hosts and their associated microbes change in concert, this is termed “coevolution.” Moreover, when two coevolving organisms also undergo speciation, this can lead to the formation of new species, i.e., co-speciation and co-diversification.

Here, we report on the opportunities in studies of marine eukaryotic host-associated microbiomes. First, we highlight our current knowledge with key examples of known ecosystem functions of host-associated marine microbes. Second, we outline ways in which comparative and experimental studies across hosts and habitats could be integrated to show how microbial symbioses at the microbiome level contribute to host evolution, resilience, and conservation strategies. There is a plethora of outstanding questions in ecology and evolution that could be addressed by expanding the phylogenetic and ecological breadth of host-associated microbiome studies, including all possible interactions throughout the microbiome. We list two questions that we believe would move the research field significantly forward, and we give specific examples of how these questions could be answered. There is strong empirical evidence and new consensus that biodiversity (i.e., the richness of species and their interactions) pervasively influences the functioning of Earth’s ecosystems, including ecosystem productivity [13,14]. However, this research has focused almost exclusively on macroorganisms. Because microbial symbionts are integral parts of most living organisms [9,15], broadening our understanding of how microbial symbionts contribute to host performance and adaptability is essential.

## How microbial symbiosis impacts marine ecosystem functioning

### Foundations of productive ecosystems

Ecosystem engineers, such as many types of corals, deep-sea mussels, and hydrothermal vent tubeworms, contribute to primary productivity and create the structural habitats and nutrient resources that are the foundation of their respective ecosystems [16]. All of these taxa engage in mutualistic nutritional symbioses with microbes. There are many examples of marine nutritional mutualisms in which microbes enable hosts to utilize resources or substrates otherwise unavailable to the host alone. Such symbioses have been described in detail in reduced and anoxic sediments (e.g., lucinid clams, stilbonematid nematodes, and gutless oligochaetes) and hydrothermal vents (e.g., the giant tube worm *Riftia pachyptila* or *Bathymodiolus* deep-sea mussels) [17]. Moreover, many foundational species of marine macroalgae are vitamin auxotrophs (for example, half of more than 300 surveyed species were unable to synthesize cobalamin), and their productivity depends on provisioning from their epiphytic bacteria [18]. Reefs often consist of scleractinian corals, one of the most well-known examples of a mutualistic symbiosis, in which the dinoflagellate alga Symbiodiniaceae supplies the coral with glucose, glycerol, and amino acids, while the coral provides the algae with a protected environment and limiting compounds (e.g., nitrogen species) needed for photosynthesis. However, this is a classic example of a mutualistic symbiosis that is sensitive to environmental disturbances, which can disrupt the fragile interactions between host and microbe. When reefs become warm and eutrophic, mutualistic Symbiodiniaceae may induce cellular damage to the host and/or sequester more resources for their own growth, thereby injuring and parasitizing their hosts [19,20]. Reef fishes, which seek homes on coral reefs, are important in fostering coral recovery in the wake of disturbance. *Epulopiscium* bacteria in the guts of surgeonfishes produce enzymes that allow their hosts to digest complex polysaccharides, enabling the host fish to feed on tough, leathery red and brown macroalgae [21]. This trophic innovation has facilitated niche diversification among coral reef herbivores. Surgeonfishes are critical to the functioning of Indo-Pacific coral reefs, as they are among the only fishes capable of consuming large macroalgae that bloom in the wake of ecosystem disturbance and suppress coral recovery [22].

Along with more standard examples of nutritional symbioses in animals, recent advances in genome sequencing technology have led to the discovery of many endosymbiotic associations in “protists” (a general term to refer to a non-monophyletic collection of unicellular eukaryotes that are not fungi or in the Plantae group) that illustrate the incorporation of various new biochemical functions, such as photosynthesis, nitrogen fixation and recycling, and methanogenesis, into protist hosts by endosymbionts [23]. Endosymbiosis in protists is widespread and represents an important source of innovation. Previously unrecognized metabolic innovations of marine microbial symbioses that are ecologically important are discovered regularly [24]. For example, Candidatus Kentron (a clade of Gammaproteobacteria found in association with ciliates) nourish their ciliate hosts in the genus *Kentrophoros* and recycle acetate and propionate, which are low-value cellular waste products from their hosts, into biomass [25]. Another interesting example is found in the anaerobic marine ciliate *Strombidium purpureum* [26]. The ciliate lives under anaerobic conditions and harbors endosymbiotic purple nonsulfur bacteria that contain both bacteriochlorophyll a and spirilloxanthin. The endosymbionts are photosynthetically active; hence, this symbiosis represents an evolutionary transition of an aerobic organism to an anaerobic one while incorporating organelles.

### Reproduction and host development

Extending beyond nutritional symbioses, microbial symbionts can alter the reproduction, development, and growth of their hosts. Specific bacterial strains in marine biofilms often directly control the recruitment of planktonic larvae and propagules, either by inhibiting settlement or by serving as a settlement cue [27,28]. For example, the settlement of zoospores from the green alga *Ulva intestinalis* onto the biofilms of specific bacteria is mediated by their attraction to the quorum-sensing molecule, acyl-homoserine lactone, secreted by the bacteria [29]. Classic examples of marine host–microbe developmental dependence include the observation that algal cultures grown in isolation exhibited abnormal morphologies [30] and the subsequent discovery of morphogenesis-inducing compounds, such as thallusin, secreted by epiphytic bacterial symbionts [31]. Bacteria are also known to influence the growth of marine plants, macroalgae, and phytoplankton by secreting phytohormones such as indole acetic acid and cytokinin-type hormones [32–34]. In the marine choanoflagellate *Salpingoeca rosetta*, both multicellularity and reproduction are triggered by specific bacterial cues, offering a view into the origins of bacterial control over animal development (reviewed by Woznica and King [35]). The benefit to the bacteria, in return, is that they receive physical space to colonize at particular points in the water column typically accessible only to planktonic microbes. Perhaps the best-studied example of intimate host–microbe interactions controlling animal development is the Hawaiian bobtail squid *Euprymna scolopes* [36]. It lives in a mutualistic symbiosis with the bioluminescent bacteria *Aliivibrio fischeri*. The bacteria are fed a solution of sugars and amino acids by the host and, in return, provide bioluminescence for countershading and predator avoidance [5]. This mutualism with microbes provides a selective advantage for the squid in predator–prey interactions. Another invertebrate example can be found in tubeworms, in which *Hydroides elegans* metamorphosis is mediated by a bacterial inducer and mitogen-activated protein kinase (MAPK) signaling in biofilms [37].

### Biofouling and microbial community assembly

Some host-associated microbes produce compounds that prevent biofouling and regulate microbiome assembly and maintenance in many marine organisms, including sponges, macroalgae, and corals [38,39]. For example, tropical corals harbor diverse bacteria in their surface mucus layer that produce quorum-sensing inhibitors and other antibacterial compounds as a defense against colonization and infection by potential microbial pathogens [1]. Epiphytic bacteria of marine macroalgae excrete a diverse chemical arsenal capable of selectively shaping further bacterial colonization and deterring the settlement of biofouling marine invertebrates such as bryozoans [32,40]. As in corals, these diverse, microbially secreted compounds include not only bactericidal and bacteriostatic antibiotics but also compounds like halogenated furanones, cyclic dipeptides, and acyl-homoserine lactone mimics that disrupt bacterial quorum sensing and inhibit biofilm formation [41]. The bacteria likely are able to utilize the carbon-rich exudates from their hosts [42,43]. For example, in the case of giant kelp, the alga emits approximately 20% of primary production as dissolved organic carbon [43]. Whereas these prior examples illustrate how the microbiomes can protect hosts from surface colonization, a similar phenomenon has also been observed internally in the shipworm *Bankia setacea*, in which symbionts produce a boronated tartrolon antibiotic thought to keep the wood-digesting cecum clear of bacterial foulants [44]. By producing antimicrobial compounds, these microbes are able to defend their niche space to prevent other organisms from crowding them out.

### Biogeochemical cycling

Host-associated microbiomes also influence biogeochemical cycling within ecosystems with cascading effects on biodiversity and ecosystem processes. For example, microbial symbionts comprise up to 40% of the biomass of their sponge hosts [45]. Through a process termed the “sponge-loop,” they convert dissolved organic carbon released by reef organisms into particulate organic carbon that can be consumed by heterotrophic organisms [2]. Along with the coral–Symbiodiniaceae mutualism, this sponge-bacterial symbiosis helps explain Darwin’s paradox, i.e., how highly productive coral reef ecosystems exist within otherwise oligotrophic tropical seas. Some sponge symbionts play a significant role in the marine phosphorus cycle by sequestering nutrients in the form of polyphosphate granules in the tissue of their host [46] and nitrogen cycling, e.g., through nitrification, denitrification, and ammonia oxidation [2,39]. Many macroalgal-associated bacteria are specifically adapted to degrade complex algal polysaccharides (e.g., fucoidan, porphyran, and laminarin [47,48]) and modify both the quality and quantity of organic carbon supplied to the ecosystem [42,49]. The sulfur-oxidizing gill endosymbionts of lucinid clams contribute to primary productivity through chemosynthesis and facilitate the growth of seagrasses (important foundation species) by lowering sulfide concentrations in tropical sediments [50]. Gammaproteobacterial symbionts of lucinid clams and stilbonematid nematodes were also recently shown to be capable of nitrogen fixation (bacterial symbiont genomes encode and express nitrogenase genes [51]), highlighting the role of symbiotic microbes in nutrient cycling in shallow marine systems.

These examples demonstrate the importance of microbial symbioses for the functioning of ocean ecosystems. Understanding symbioses with this same level of detail in the context of complex communities (i.e., whole microbiomes) remains ripe for exploration and, indeed, requires a more integrated framework from the fields of microbiology, evolutionary biology, community ecology, and oceanography. Individual taxa within the microbiome may help hosts withstand a wide range of environmental conditions, including those predicted under scenarios of climate change. Next, we explore two different avenues of how interdisciplinary collaborations could advance this line of research.

## Two example outstanding questions: The influence of microbiomes in a changing ocean

### I. How can host-associated microbiomes influence host adaptation in a changing ocean?

**Fig 1:**
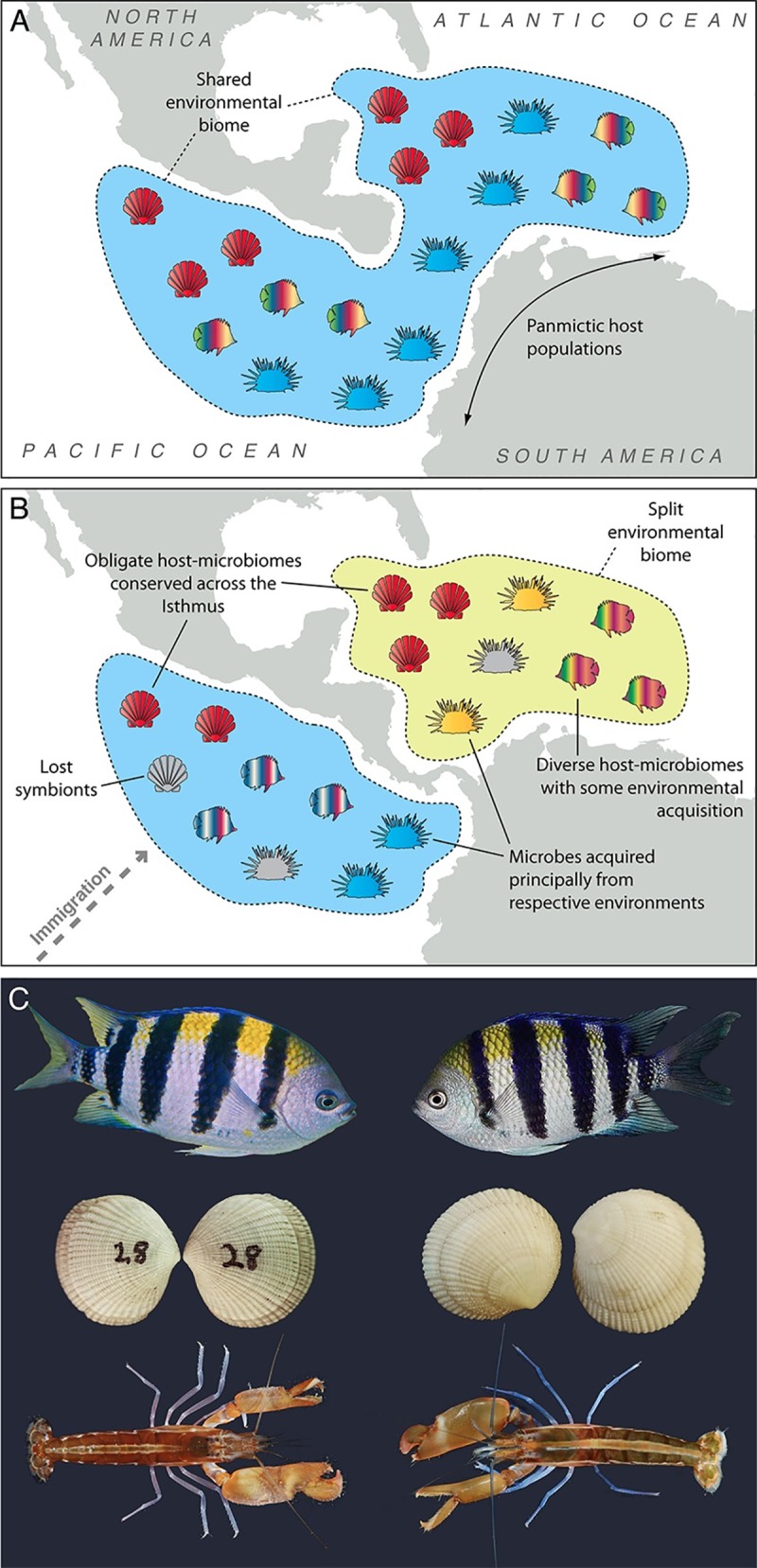
The formation of the Isthmus of Panamá split an ocean into two, creating a natural experiment to explore general processes of host–microbe evolution. (A) Panmictic populations of hosts and their microbiomes living under similar environmental conditions became (B) physically isolated when the land bridge formed between North and South America. Well-calibrated phylogenetic data are available for marine animal hosts such as clams, fishes, porcelain crabs, snapping shrimps, and urchins. (C) Example of sister species isolated by the Isthmus of Panamá (left: Eastern Pacific, right: Caribbean): fish, *Abudefduf saxatilis* and *Abudefduf troschelii*; clams, *Ctena mexicana* and *Ctena distinguenda*; and shrimps, *Alpheus panamensis* and *Alpheus formosus*. *Photo credit*: *fish*, *Ross Robertson* (A. saxatilis) *and Ettore Balocchi* (A. troschelii); *clams*, *Laetitia G*. *E*. *Wilkins and Benedict Yuen; shrimps*, *Arthur Anker*.

Global change creates new niches and conditions to which organisms must adapt. Whether and how marine species adapt to change may depend on their microbiomes. Host-associated microbes can be treated as extended host phenotypes if host and microbe show a concerted adaptive response [17]. Geological events have played central roles in driving evolution [52]. Studying them can provide great insight into the processes of adaptive evolution [53]. The formation of the Isthmus of Panamá, for example, represents a natural evolutionary experiment. Until relatively recently (on a geological time scale), the Tropical Eastern Pacific (TEP) and Caribbean Sea were connected, and marine life could mix freely [54,55]. Before the Isthmus formed, the Caribbean and the TEP shared a homogenous, nutrient-rich biotic realm [54]. Over millions of years, the rising land bridge blocked interoceanic currents, causing the Caribbean to become oligotrophic and allowing the great tracts of reefs and seagrasses we know today to proliferate [56]. Conversely, the Pacific coast continued to be dominated by nutrient-rich ecosystems [57,58], and reefs remain rare and seagrasses all but nonexistent. Within this major environmental and ecological divergence, once-contiguous populations became isolated and followed distinct eco-evolutionary paths [55]. Today, all major groups (except corals) have representative species pairs that were split by the Isthmus, and most have examples distributed across their respective clades [55,59]. The Isthmus system therefore offers a remarkable opportunity to explore drivers and processes of speciation, diversification, and adaptation with a replicated suite of taxonomic and functional host–microbiome assemblages, often with well-calibrated phylogenetic support (Fig 1). We can explore questions of parallel versus differential evolution between hosts and their microbiomes, reveal general processes of adaptive evolution (e.g., loss/gain of genes in microbial genomes), and unveil the relative contribution of vertical and horizontal transmission in marine host communities [60].

Vertical transmission of symbiotic microbes to the offspring is expected to stabilize the association between a given host and microbe, thereby making phenotypic traits of the host–microbe interaction potentially heritable. This stabilization could, in turn, drive adaptive evolution of host–microbe interactions if it allows host populations to adapt to new ecological niches or persist in a changing environment [15,61]. In contrast, horizontal transmission of symbiotic microbes generally requires some form of selective host filtering of beneficial symbionts and/or host sanctioning of detrimental symbionts and cheaters in order to align host and microbial interests and coordinate their adaptive responses [62]. Indeed, some corals can expulse their Symbiodiniaceae algal symbionts in exchange for a set that is better adapted to a given condition [63]. Consequently, horizontally acquired symbionts have more opportunity to exchange genes with environmental and/or free-living conspecifics and can thereby increase their adaptive potential [64]. The acquisition of novel symbionts has been postulated as a form of phenotypic plasticity that could potentially assist foundational seagrass and macroalgal species acclimatize to a changing climate [65]. Studying taxonomic, genomic, and functional microbial compositions in combination with the phylogenies and habitats of their hosts either side of the Isthmus will help identify which microbes are tightly associated with the host, their mode of transmission, and their genomic features [66,67]. The Isthmus system is not an isolated example. The relatively recent emergence of oceanic islands (e.g., Marquesas, Hawai’i, or the Galapagos Islands), for example, can be used to reveal processes of coevolution in marine hosts and their associated microbes [68]. Indeed, natural events like these have been instrumental in resolving ecological and evolutionary processes in multicellular life. So long as testable predictions and hypotheses are constructed, we propose they can offer significant insights into host–microbiome assemblage evolution and ecosystem functions.

### II. How can we use marine host-associated microbiomes to inform conservation?

Human activities are directly affecting the composition of natural microbiomes. Examples include the introduction of pathogens as well as non-native species and their microbial symbionts [69] and environmental contamination with antimicrobials in offshore farms [70]. Conventional aquaculture practices can promote high numbers of diverse bacteria on farmed hosts (some of them symbionts and some pathogens), that, in combination with the use of antibiotics, can develop into hotspots for horizontal gene transfer [71] and consequent dissemination of antibiotic resistance [70]. The composition of host-associated microbiomes may also be modified by other stressors, such as elevated seawater temperatures associated with global climate change or the locally discharged water from power plants [72], oil spills [73], and contamination with heavy metals from mining activities [74], with potential effects on host biology. Microbial-based mitigation strategies can be focused on specific contaminants or threats (e.g., oil-degrading bacteria, heavy metal immobilization, biological control of pathogens) or more broadly, based on the maintenance or improvement of the host health. For example, corals exposed to high temperatures were significantly more resistant to bleaching when inoculated with a consortium of microorganisms isolated from native healthy host corals [75]. Jin Song and colleagues (2019) summarized and discussed examples of successful probiotics used to promote animal health and conservation in the wild [76]. Such mitigation strategies, like these that make use of host-associated microbiomes by direct bioaugmentation (i.e., enriching the environment with specific microbes) or through the biostimulation of specific metabolisms to enhance host resistance and recovery, are promising but remain rare and in their infancy.

Effective microbially based mitigation will benefit from a thorough understanding of the identity and physiology of beneficial microbes and the attributes of healthy microbiomes, although microbial mitigation via bioaugmentation may only be effective for horizontally acquired symbionts. To this end, during the most successful trials of microbiome engineering, scientists have considered niche-specific traits and the manipulation of stable and native groups rather than the use of generic microbial cocktails [77]. Nevertheless, manipulative approaches can succeed even without knowing the detailed mechanisms a priori, as long as a rigorous experimental design is applied, which can eventually lead to the discovery of key strains and mechanisms [39,78].

From the perspective of applied ecosystem recovery, the most promising focal organisms for such bioaugmentation projects are keystone and foundational organisms and their associated microbiomes, as such efforts can cascade throughout the ecosystem by enhancing recovery of the central food source and biological niche within the habitat. Thus, efforts to quantify and compare the net effects of microbiome functions across multiple hosts and contexts (e.g., health status, life stage, and habitat) are critical to advancing our understanding of the roles of microbiomes for threatened hosts and ecosystems [19,79]. The fact that there are microbiomes specific to different developmental stages in tropical corals suggests that microbiomes may serve distinct, specific roles throughout host life cycles [80]. Environmental stressors can compromise or eliminate mutualistic microbe species that need to be replaced by beneficial, or at least neutral, microbes to passively prevent the spread of diseases. Incubation experiments and mesocosm setups are urgently needed to study the individual and interactive effects of increasingly common disturbances such as increased temperature, changes in partial pressure of carbon dioxide and acidification, nutrient enrichment, and physical damage on foundation and keystone species’ microbiomes [81]. The main goal for future studies of microbiomes in conservation biology is understanding the degree to which important functional roles can be maintained in nonoptimal environmental conditions and whether diverse communities of transient microbes may allow hosts to broaden their realized ecological niche [82,83]. Describing and understanding the organizing principles of microbiome assembly and maintenance is critical for developing effective microbial-based mitigation strategies [84,85]. Studying shifts in microbiome taxonomic composition and functional diversity in organisms that experience drastic seasonal or thermal shifts (e.g., temperate organisms or species living in intertidal zones) will help identify these principles [86,87]. For example, the microbiome of the temperate coral *Astrangia poculata* resembles a diseased tropical coral microbiome in the winter months, during host quiescence, and it transitions in the spring to a community dominated by taxa that continue to be present throughout the year [88]. This seasonal shift represents an opportunity to identify the molecular basis of microbiome assembly within an animal host. Such complex interactions among microbial species and their hosts can be informed by theory and empirical generalizations in community ecology developed primarily from studies of macroorganisms, including succession, community assembly, metacommunities, multitrophic interactions, disturbance, and restoration [84–86].

## Integration of information across hosts for an ecosystem-level understanding of the roles of microbial symbionts

Future progress in microbial symbioses research—and, indeed, in our understanding of ecosystem functioning generally—depends on adopting a microbiome perspective and expanding the scope of inquiry beyond single host taxa. First, this will require a broad comparative approach to identify similarities and differences across marine host species within a phylogenetic framework, especially with respect to their physiologies, microbiome profiles, and habitat distributions. Second, studies of terrestrial hosts and microbiomes can inform research priorities and generate hypotheses to be tested in marine environments [61,89]. Thus, we see great value in building a framework of broad collaborative networks. Collaborative efforts are more sustainable, and ultimately more productive, if we credit online resource generators, share data and workflows, and acknowledge others [90].

Identifying the factors that promote the contribution of microbial symbionts to host adaptability is fundamentally important to understanding ecological and evolutionary processes as well as predicting the response of populations, species, and communities to a changing environment. Key events (e.g., formation of the Isthmus of Panamá) can provide model systems to test hypotheses about the roles of marine host-associated microbiomes for ecosystem functioning. The biggest payback will likely come from a focus on taxa that have disproportionately large roles in the ecosystem, including dominant, foundational, and keystone species. We recommend special focus on how horizontally transferred microbes play critical roles in the hosts’ ability to respond to environmental change because we predict that these types of symbionts may be able to adapt quicker than their vertically transmitted counterparts. Together, these research directions will enhance our ability to predict how climate change, invasion by non-native species, food web disruption, and environmental contamination will affect species and inform practical strategies for directly assisting marine conservation in novel ways. For example, understanding the influence of the microbiome on host development and function will ultimately help prioritize management decision.

## Abbreviations

MAPK: mitogen-activated protein kinase
TEP: Tropical Eastern Pacific
